# HIV cure trial mergers: Spotlighting the epigenetics of latency reversal

**DOI:** 10.1016/j.ebiom.2022.104012

**Published:** 2022-04-19

**Authors:** Paul W. Denton

**Affiliations:** Department of Biology, University of Nebraska Omaha, Omaha, NE, USA

## Commentary

Developing a scalable approach to cure HIV-1 infection remains a high priority.^1^ In recent years, pivotal advances have pushed the field toward such a cure – which could be realized as complete virus eradication or sustained antiretroviral therapy-free HIV-1 remission.^2^ Many of these advances have arisen from small, interventional clinical trials.^3^ However, a notable challenge in the field of HIV cure research has been the lack of relevant control arms in these clinical studies.^4^

In this issue of *eBioMedicine*, Oriol-Tordera et al. have taken a unique and clever approach to help tackle this limitation.^5^ Their new manuscript merges data from two distinct interventional clinical trials where the single-arm BCN02 study (NCT02616874) is the “experimental arm” and the single-arm REDUC study (NCT02092116) is the “control arm”.^6^^,^^7^ This merging of trials is not implying that BCN02 and REDUC are perfectly matched trials. For example, BCN02 rolled in participants from BCN01 (NCT01712425) where all individuals were enrolled with confirmed acute/recent HIV-1 infection (under 6 months from estimated HIV-1 acquisition).^8^ This is a notable distinction from the REDUC study where participants were required to have been receiving suppressive antiretroviral therapy for a minimum of 1 year prior to enrollment. Also, differing trial site locations and study timeframes resulted in inherent differences in sample handling. Nevertheless, samples from the REDUC study were quite valuable for use by Oriol-Tordera et al. This is because, the REDUC samples originated from participants whose only intervention beyond antiretroviral therapy was treatment with romidepsin.^7^ In contrast, the BCN02 trial combined romidepsin treatment with re-vaccination with the HIVconsv prime/boost therapeutic vaccination approach originally administered in BCN01.^6^ Because histone deacetylase inhibitors (e.g. romidepsin) impact chromatin arrangement, they are being evaluated as latency reversal agents to reactivate quiescent HIV-1 proviruses with the expectation that the cell with reactivated virus will be killed via immune-mediated clearance or through viral cytopathic effects. In the new manuscript, the REDUC samples serve as a surrogate romidepsin-only control arm for the transcriptomics and whole-genome DNA methylation analyses presented.^5^ Inclusion of the romidepsin-only controls helps to disentangle the effects of the romidepsin intervention from the impacts of the vaccination regimen utilized in the BCN02 study.

A key hypothesis of the new study was that epigenetic imprints could serve as biomarkers of HIV control during antiviral therapy interruption. To test this, the investigators performed extensive transcriptomics and DNA methylation analyses on samples from both trials and then performed gene set enrichment analyses to help identify differences associated with vaccination plus romidepsin versus romidepsin alone. Samples collected during the treatment interruption phase of the study were stratified according to whether individuals maintained a viral load of <2000 HIV-1 RNA copies/mL for more than four weeks or not. The authors report that DNA methylation patterns assessed before antiretroviral treatment interruption may allow for predicting time to viral rebound following treatment interruption. One such broad pattern was that hypomethylation in regions of repressed chromatin at the time of intervention was associated with delayed viral rebound following antiretroviral treatment interruption.^5^ It remains to be seen whether the described methylation signatures can be used prospectively to predict antiretroviral therapy interruption outcomes.

Bridging methylation and gene expression patterns in the context of intervention HIV cure-related clinical trials, especially trials that use latency reversal approaches, is quite innovative and important. Yet, several open questions remain. One such open question is: Can findings from the epigenetic and transcriptomic analyses be validated in *ex vivo* or *in vitro* experiments? For example, the data presented indicate that *PRKCQ* methylation levels are negatively correlated with cell-associated HIV-1 RNA as well as HIV-1 proviral levels. This is noteworthy because *PRKCQ* is known to be relevant in HIV-1 latency, including in the context of latency reversal approaches.^9^ Yet, sample availability precluded further investigation into this and other notable associations discovered in the study. Thus, future trials could benefit from designs that ensure sufficient materials are biobanked and allocated to discovery as well as validation experiments. Another open question is: What are the specific epigenetic signatures at HIV integration sites? It should be noted that, Oriol-Tordera et al. designed their study to identify host DNA methylation changes, not changes in DNA methylation of viral genomes. Nevertheless, the authors were able to identify associations between known HIV integration sites and viral reservoir measures (e.g. BACH2^10^ methylation levels correlated with cell-associated HIV-1 RNA). The partial answers provided to these as well as other open questions are hypothesis generating and will guide next steps in this area.

In conclusion, Oriol-Tordera et al. have provided a valuable resource for the HIV cure research community. The DNA methylation signatures identified in this latency reversal study point to potential biomarkers of antiretroviral therapy interruption outcomes. Furthermore, the authors have provided a blueprint for the successful merging of independent clinical trials to help overcome challenges associated with single-arm designs.

## Contributors

PWD wrote this commissioned commentary.

## Declaration of interests

The author declares no conflicts of interest.
